# Meeting report: the inaugural muscle biology and cachexia conference at the University of Houston, May 18-20, 2025, Houston, Texas, USA

**DOI:** 10.3389/fphys.2025.1711795

**Published:** 2025-10-30

**Authors:** Jingjuan Chen, Marco Brotto, Radbod Darabi, Ashok Kumar

**Affiliations:** ^1^ Department of Orthopaedic Surgery, Duke University, Durham, NC, United States; ^2^ Bone-Muscle Research Center, College of Nursing and Health Innovation, The University of Texas at Arlington, Arlington, TX, United States; ^3^ Institute of Muscle Biology and Cachexia, University of Houston, Houston, TX, United States; ^4^ Department of Pharmaceutical and Pharmaceutical Sciences, University of Houston College of Pharmacy, Houston, TX, United States

**Keywords:** bone-muscle crosstalk, bone, muscle, cachexia, molecular and signaling pathways, muscle physiology and disease

## Abstract

In May 2025, the University of Houston (UH) hosted the inaugural Muscle Biology and Cachexia conference, organized by Drs. Ashok Kumar and Radbod Darabi. The conference attracted nearly 300 participants, including established scientists, early-career researchers, and students from across the United States, Canada, Italy, Singapore, and Turkey. Research was presented through a combination of oral presentations and poster sessions. The conference was driven by the increasing interest in skeletal and cardiac muscle biology and cancer cachexia among institutions at the Texas Medical Center and surrounding universities. It served as a platform to promote knowledge exchange and foster collaboration within this growing scientific community. The conference was supported by the UH College of Pharmacy (UHCOP), Division of Research (DOR), Drug Discovery Institute (DDI), and the Department of Pharmacological and Pharmaceutical Sciences (PPS). In conjunction with the conference, UH announced the formation of the Institute of Muscle Biology and Cachexia (IMBC). The IMBC aims to strengthen collaborative research efforts and enhance understanding of the molecular and signaling pathways that regulate muscle physiology and disease.

## Introduction

The conference focused on skeletal and cardiac muscle biology with a plethora of topics including muscle stem cell biology, skeletal muscle physiology and pathology in exercise and disease conditions, and the role of immune system. The goal of the conference was to explore how different signaling pathways, macromolecules, extracellular matrix, and various cell types contribute to muscular system health, as well as their possible pathogenic role in muscle disorders and cachexia. The conference program included two keynote speakers, 46 invited speakers, 21 short talks, and 63 posters over the course of 3 days. Scientific sessions included: Muscle progenitors, regeneration, and rhabdomyosarcoma; Ageing and sarcopenia; Mechanisms of cancer cachexia I & II; Cardiac biology and disease; Muscle signaling and metabolism; Exercise physiology; and Muscle diseases and therapies I & II. The exciting new perspectives in each section broadened our understanding of the muscular system. Some of the highlights of the sessions are included in the following sections.

### Nuclear factor-kappa B (NF-κB) signaling in skeletal muscle

Dr. Denis Guttridge (Medical University of South Carolina, South Carolina, United States), one of the two keynote speakers, highlighted an updated regulatory network of the NF-κB signaling as one of the key players in the skeletal muscle wasting during cancer cachexia. Dr. Guttridge’s work demonstrated how NF-κB can be exploited to help alleviate muscle loss due to cancer cachexia. In the cancer environment, tumor cells can secrete the soluble factor GDF15 (Growth and Differentiation Factor 15), which signals to the macrophages and suppress the transforming growth factor β-activated kinase 1 (TAK1)-NF-κB axis for the removal of tumor cells (Ratnam et al., 2017). Interestingly, different cell types in the muscle adapt differently to the persistent NF-κB activation during cancer cachexia. While muscle stem cells (MuSCs) and fibro-adipogenic progenitors (FAPs) have elevated NF-κB level due to the local inflammation cues in cachexia, inhibition of NF-κB signaling in MuSCs alleviates the loss of muscle and body mass during cachexia. However, NF-κB inhibition in FAPs only leads to a reduced immune infiltration but not a rescue in muscle mass or body mass, suggesting that different cell types (MuSCs and FAPs) contribute to different aspects in skeletal muscle maintenance during cancer cachexia (Pryce et al., 2024).

Rhabdomyosarcoma (RMS) is the most common soft tissue sarcoma in children that arises in or near skeletal muscle beds. RMS develops from mesenchymal cells that have failed to fully differentiate into myocytes of skeletal muscle (Xia et al., 2002). Interestingly, while most cancer cell lines are sensitive to the inhibition of NF-κB pathway, the RMS cell lines can still proliferate and survive under NF-κB inhibition with the aid of MyoD transcription factor that maintains the cells in a partially differentiated state. Dr. Guttridge’s work further demonstrated that repression of MyoD induces the dedifferentiation of RMS and sensitizes them for apoptosis following NF-κB inhibition (Oles et al., 2025).

Besides Dr. Guttridge’s research, several other invited speakers also contributed to the wholistic understanding of the NF-κB signaling in cancer cachexia. Dr. Serkan Kir (Koç University, Turkey) showed that EDA2R (Ectodysplasin A2 Receptor) and OSM (Oncostatin M) were highly upregulated in muscle from tumor-bearing mice. Interestingly, Type IIb myofibers have higher transcript abundances for *Eda2r* and atrogenes (*Fbxo32* and *Trim63*), which might be the reason Type IIb myofibers are prone to undergo atrophy in cancer cachexia. Mechanistically, EDA2R promotes muscle atrophy by activating the non-canonical NF-κB signaling pathway which involves p52/RelB dimers (Domaniku-Waraich et al., 2024; Bilgic et al., 2023). The laboratory of Dr. Mikhail Kolonin (McGovern Medical School, UTHealth, Houston, Texas, United States) explored the molecular mechanisms of muscle dysfunction with the administration of GLP1-receptor agonists for weight loss. The study demonstrated that blunting the IL-6 signaling axis in skeletal muscle can rescue muscle dysfunction while still preserving the body weight loss function of GLP1-receptor agonists such as Liraglutide. Moreover, studies by Dr. Joseph Rupert from the same group showed that inactivation of IL-6 receptor expression in skeletal muscle cells can reduce cachexia resulting from pancreatic cancer as well as suppress cancer progression.

### Atrogenes and skeletal muscle atrophy

Dr. Marco Sandri (University of Padova, Italy) was another keynote speaker who presented his recent research related to mitochondrial network and its correlation with muscle diseases, as well as the role of proteostasis in muscle mass maintenance. Dr. Sandri highlighted that while mitochondrial hyperconnectivity usually leads to calcium dysregulation which leads to muscle dystrophy, mitochondrial fragmentation leads to the production of reactive oxygen species (ROS), leading to muscle atrophy and cell death. More importantly, as we advance into the new era of transcriptomics analysis with the aid of machine learning, Dr. Sandri gave an elegant example of the identification of an uncharacterized gene *Mytho* (Macroautophagy and YouTH Optimizer) to illustrate that there are still many unknown territories awaiting further study (Leduc-Gaudet et al., 2023). Another discussed research was the cell-cell crosstalk in the cachexia through the signaling axis of muscle derived Noggin to inhibit the BMP signaling in neuronal cells at the neuromuscular junction, which further exacerbates the muscle wasting in cachexia (Sartori et al., 2021).

To further elucidate the role of atrogenes in muscle atrophy, Dr. Nora Hosny (University of Minnesota, United States) demonstrated that cardiomyocytes exhibited impaired NFAT signaling due to cytoplasmic retention of NFAT and reduced transcriptional activity, resulting from decreased levels of calcineurin A. She further proposed that Atrogin-1 (*Fbxo32*) mediates the degradation of calcineurin A in cachectic cardiomyocytes. Dr. Jason Doles (Indiana University, Indiana, United States) discussed the tumor-muscle interactions for the development of better therapeutics in cancer patients. He presented results demonstrating that muscle-specific MuRF1-knockout (KO) blunts the KPC-induced muscle atrophy and extends survival despite tumor burden in mice. Moreover, he showed that the conditioned medium from cachectic myotubes can promote tumor growth, suggesting an intricate crosstalk between skeletal muscle and tumor (Neyroud et al., 2023).

### The role of MuSCs and stromal cells in skeletal muscle health and disease

As the resident stem cell population which helps repair the muscle, satellite cells were another focus at the conference. Dr. Atsushi Asakura (University of Minnesota, Minnesota, United States) presented his recent work on endothelial cell-muscle stem cell crosstalk. He presented results about the signaling axis of Jag2-Notch signaling from endothelial cells to MuSCs. *Jag2* hypo-morphism in ECs leads to impaired myogenic differentiation due to dysregulated cis-inhibition of the Notch signaling pathway. Dr. Hamed Jafar-Nejad (Baylor College of Medicine, Houston, Texas, United States) underscored the often-overlooked role of post-translational modifications of the Notch receptors. They modeled limb-girdle muscular dystrophy by generating satellite cell-specific O-glucosyltransferase I (*Poglut1*) KO mice. Mice lacking *Poglut1* in myogenic progenitors demonstrated severe muscle dystrophy with reduced *Pax7* expression and abnormality in the extracellular matrix (ECM). They further uncovered that NOTCH1, NOTCH2 and NOTCH3 are targets of POGLUT1, further corroborating the importance of NOTCH signaling in the maintenance of muscle stem cell pool (Cho et al., 2025). In keeping the theme with the ECM, Dr. Christopher Fry (University of Kentucky, Kentucky, United States) demonstrated that COL1A1 and COL1A2 are the most upregulated types of collagens during muscle overloading. He showed that a subset of Periostin^+^ myofibroblasts highly upregulate lysyl oxidase (*Lox*) which leads to elevated collagen crosslinking and thereby decreased ECM plasticity for the remodeling required for the adaptation to muscle hypertrophy. Conditional deletion of the *Lox* in the Periostin^+^ cells leads to a more favorable ECM environment for muscle hypertrophy. Dr. Feng Yue (University of Florida, Florida, United States) demonstrated the importance of the nutrient sensing pathway during myogenic differentiation of the muscle satellite cells. He demonstrated that cAMP response element-binding protein (CREB)-regulated transcription coactivator (CRTC) manifests dynamic translocations in the muscle stem cells at different cellular states, which can be correlated with the energy state of the cells.

Additionally, the roles of the stromal cells in the skeletal muscles such as FAPs and immune cells were also discussed. Dr. Laszlo Nagy (School of Medicine, Johns Hopkins University, Florida, United States) demonstrated an atlas of the immune cells during muscle regeneration using multi-omics (Patsalos et al., 2024). Dr. Jyoti Jaiswal (Children’s National Research Institute, Washington DC, United States) showed that Annexin A2 was upregulated in dysferlinopathy. Through single cell RNA-seq, they showed that *Timp1* ^+^ and *Postn* ^+^ FAPs are pathogenic which leads to aberrant adipogenesis in muscle dystrophy. Dr. Darko Bosnakovski (University of Minnesota, Minnesota, United States) demonstrated the metalloproteases (MMPs) were the culprit in Facioscapulohumeral muscular dystrophy (FSHD), by using a *Dux4* overexpression mouse model to mimic the FSHD. FAPs and macrophages were the major cell types producing the MMPs, and pharmacological inhibition of MMPs can help attenuate the disease progression in FSHD. In another disease model, Dr. Terence Ryan (University of Florida, Florida, United States) showed in Peripheral Artery Disease (PAD), the excessive accumulation of intramuscular adipose tissue was due to the increased adipogenic activities from FAPs. Overexpression of the master adipogenic regulator *Ppar*γ in the FAPs exacerbates the muscle regeneration under ischemic injury, while KO of *Ppar*γ in the FAPs improves the muscle recovery.

### Macromolecules in the homeostasis of skeletal muscle

Another highlight of the conference was the contribution of macromolecules to muscle homeostasis. Drs. Pankaj Singh (University of Oklahoma, Oklahoma, United States) and Ravi Singh (University of Houston, Texas, United States) both revealed that ketone bodies play an important role in muscle physiology, where Dr. Pankaj Singh showed that cachectic potential of pancreatic cancer cells is attenuated by ketone bodies treatment. Dr. Ravi Singh showed that an alternative splicing isoform of *Mef2d* modulates the expression of the key enzymes for utilization of ketone bodies, and the *Mef2d*α2 KO mice have reduced ketone body metabolism (Kumar et al., 2025). Dr. Jianjie Ma (University of Virginia, Virginia, United States) continued his quest on the application of the pro-regenerative protein MG53 to various disease conditions. Similarly, Dr. Jingsong Zhou (University of Texas Arlington, Texas, United States) demonstrated the potential of MG53 in the alleviation of the disease progression of ALS mice model. Several speakers highlighted the role of lipids on skeletal muscle. Dr. Marco Brotto (University of Texas Arlington, Texas, United States) showed that PGE2 can be secreted from bones and exert its action on skeletal muscle. He also showed a few developed models at his center using targeted Lipidomics and Metabolomics platforms for clinical and translational applications (Awad et al., 2024; Lyssikatos et al., 2023; Wang et al., 2020; Wang et al., 2017). Dr. Blake Rassmussen (University of Texas Heath San Antonio, Texas, United States) showed that short-term muscle disuse in human can lead to the decrease of phosphatidylinositol but an increase of phosphatidylglycerol and diacylglycerol (Kilroe et al., 2025). Dr. Reshma Taneja (National University of Singapore, Singapore) showed that phospholipase A2 levels were increased in alveolar RMS 3D tumor sphere compared to 2D cell culture, with increased lipid droplet formation (Gupta et al., 2025). These studies highlighted the dynamic regulation of lipid metabolism in tumor cells.

### Calcium signaling

The important role of calcium for muscle contraction was underscored by Dr. George Rodney (Baylor College of Medicine, Texas, United States) who showed excessive leak of SR calcium underlies the pathology of malignant hyperthermia. Dr. Erin Seifert (Thomas Jefferson University, Pennsylvania, United States) further showed that phosphate carrier SLC25A3 can regulate mitochondrial calcium handling. Dr. Andrew Judge (University of Florida, Florida, United States) highlighted the important role of the complement system in the progression of cancer cachexia (D'Lugos et al., 2025). Dr. Gustavo Nader (Pennsylvania State University, Pennsylvania, United States) showed that methylation of the rRNA can alter the translation capacity of the ribosomes, which contributes to the anabolic effects of cancer cachexia. Lastly, one of the few translational studies that bridge the research into clinical trials was the use of carnosine in the treatment of the PAD, which showed promising results. Dr. Shahid Baba (University of Louisville, Louisville, United States) carried out this trial after their initial characterization of low carnosine levels in PAD patients as a no-mechanism based therapy. Dr. Mattia Quattrocelli (University of Cincinnati, Cincinnati, United States) also made a similar discovery that carnosine has anti-sarcopenic effects. Additionally, as a member of the CPAM (Center for Precision Animal Models), Dr. Matthew Alexander (University of Alabama at Birmingham, Alabama, United States) showcased that a patient specific *Vma21* mutation can be modeled, and he also demonstrated that an artificial intelligence tool, mediKanren, can be used for unbiased screen of the drugs that can target the gene of interest.

### Benefits of exercise on inflammation and metabolism

Dr. Melissa Markofski (University of Houston, Texas, United States) pitched a new notion that instead of calling inflamm-aging, it should be inflamm-inactivity, by showing that inactivity was the reason behind the inflammation and muscle dysfunction. Dr. David Harrison (Boston Children’s Hospital, Massachusetts, United States) corroborated with the view by showing that patients with a single ventricle instead of both left and right ventricle in normal people, benefited from moderate exercise and should be encouraged to do so, contrary to the conventional perspective that these patients should not engage in physical activities. Dr. Zhen Yan (Virginia Tech Roanoke, Virginia, United States) provided molecular evidence to support that AMPKα2 (Thr172) phosphorylation is crucial for mitochondrial adaptation in exercise, but not for improved exercise capacity. Dr. Marc Hamilton (University of Houston, Texas, United States) demonstrated that while small in mass, contractile activity from soleus muscle goes a long way for the whole-body metabolic benefit (Hamilton et al., 2022). Dr. Chunru Lin (University of Texas MD Anderson, Texas, United States) showed that exercise-induced circulating neurotrophic factors can protect smooth muscle integrity and prevent tumor-associated lymphangiogenesis.

### New insights into muscle biology

Dr. Taejeong Song (University of Arizona, Arizona, United States) showed that fast myosin binding protein C is essential for fast twitch myofiber function and can exacerbate the age-related muscle dysfunction. Dr. Shihuan Kuang (Duke University, North Carolina, United States) utilized elegant experimental designs to demonstrate that the carnitine palmitoytransferases play an important role in the regulation of the acetyl-CoA pool in muscle stem cells. Dr. Zheng (Jake) Chen (UT Health Houston, Texas, United States) demonstrated that RORs (retinoic acid-related orphan receptor alpha) can regulate mitochondrial metabolism in a circadian-dependent manner. Dr. Vihang Narkar (McGovern Medical School, Texas, United States) showed that estrogen-related receptors (ERRs, a family of orphan nuclear receptor transcription factors) not only are the determinants of oxidative fiber types but also play an important role in muscle regeneration in various muscle diseases, especially Duchenne muscular dystrophy (Nguyen et al., 2025). Dr. John Lawler (Texas A&M University, Texas, United States) gave a presentation about the importance of the mu-splice variant of the nitric oxide synthase in muscle hypertrophy and atrophy. Dr. Robert Schwartz (University of Houston, Texas, United States) demonstrated that N-terminus of Serum Response Factor’s MADS box (STEMIN) and the YAP mutant (YAP5SA) worked by inducing the Yamanaka factors to increase cardiomyocyte regeneration. They showed that the microRNAs that were secreted through the exosomes might be exploited to block cardiomyocyte cell death (Bejar et al., 2024; Xiao et al., 2022). Dr. Tamer Mohamed (Baylor College of Medicine, Texas, United States) showed through scRNA-Seq that CD36^+^ cardiomyocytes are primed for proliferation and fatty acid binding protein 5 (FABP5) and peroxisome proliferator-activated receptor δ (PPARδ) work synergistically to safeguard the proliferation of cardiomyocytes.

### Cures for muscle diseases, from bench to clinics

Besides the basic research, the therapeutic application is always inspiring. Dr. Shih-Yin Tsai (National University of Singapore, Singapore) showed that eukaryotic translation initiation factor 4E (eIF4E) binding protein 1 (EIF4EBP1 or 4E-BP1) activation proved to be beneficial in sarcopenia, where the proteostasis is disrupted. Dr. Min Li (University of Oklahoma, Oklahoma, United States) demonstrated that zinc transporters were upregulated in several cancers and targeting zinc transporters in combination with surgical resection of pancreatic cancer can have better clinical outcomes. Dr. Paola Costelli (University of Torino, Italy) demonstrated that IL-4 can be used to counteract cancer cachexia (Iaia et al., 2025; Costamagna et al., 2020). Dr. Longhou Fang (Methodist Research Institute, Texas, United States) elegantly demonstrated the multi-modal action of AIBP protein in angiogenesis and hematopoietic development, which may have therapeutic potential. Dr. Andrea Bonetto (University of Colorado, Colorado, United States) characterized the prevalence of cachexia in patients with head and neck cancer and showed that the patients undergoing surgery have high incidence of cachexia, but females were less susceptible. They also developed a mouse model to study the disease progression of head and neck cancer. Dr. James Carson (Texas A&M University, Texas, United States) explored the long-term effects of chemotherapy on skeletal muscles, which was often neglected in clinics.

## Conclusions and future perspectives

The inaugural Muscle Biology and Cachexia Conference at the University of Houston brought together a distinguished group of renowned speakers who presented novel and impactful data across multiple domains, including muscle biology, cachexia, and neuromuscular diseases. A key focus of the conference was the complex interaction between cancer cells and the host immune response that drives cachexia in cancer-bearing individuals. There was also extensive discussion on the pathophysiological mechanisms underlying muscular dystrophy and other neuromuscular disorders. Additionally, the role of exercise and metabolic reprogramming in skeletal and cardiac muscle under various physiological and pathological conditions generated considerable interest and dialogue. The conference provided a platform for in-depth discussions on recent discoveries and the future direction of research in these evolving fields.

Importantly, the event fostered meaningful collaboration opportunities among researchers from diverse institutions. A shared consensus emerged on the need to integrate advanced genomic and proteomic technologies to investigate transcriptional, post-transcriptional, and post-translational changes in distinct cell populations within skeletal and cardiac muscle, particularly in disease models. Further research is also needed to elucidate cell-cell interactions in both healthy and diseased states. Publicly available genomic and proteomic datasets were highlighted as valuable tools for deepening our understanding of the molecular mechanisms that regulate muscle function and disease progression. A curated list of online resources to explore gene, protein, and metabolite expression in the context of exercise and muscle injury is provided in Table 1.

**TABLE 1:** Summary of the online resources to explore gene/protein/metabolite expression.

Human/Mouse exercise transcriptome		
GEPREP	http://www.geprep.org.cn/home/index/	
METAMEx	https://www.metamex.eu/app/metamex	Pillon et al. (2020)
ExerGeneDB	https://exergenedb.com/	Pan et al. (2025)

Transcriptome, metabolome and proteome after exercise		
MoTrPAC	https://motrpac-data.org/	Sanford et al. (2020)

Single cell RNAseq dataset in muscle tissues in different conditions		
Myoatlas	https://research.cchmc.org/myoatlas/	Petrany et al. (2020)
EMBL-EBI Single Cell Expression Atlas	https://www.ebi.ac.uk/gxa/sc/home	
HLMA	https://db.cngb.org/cdcp/hlma/	Lai et al. (2024)
SCSMRD	https://scsmrd.fengs-lab.com/	FENG et al. (2023)
scNavigator: beta	https://artyomovlab.wustl.edu/scn/	

The conference concluded with awards recognizing the best oral and poster presentations by trainees, an initiative aimed at encouraging young scientists in the field. Dr. Ashok Kumar delivered the closing remarks, expressing his sincere appreciation to all speakers, participants, and sponsors, and shared his enthusiasm for future editions of the Muscle Biology and Cachexia conference at the University of Houston.

## Data Availability

The original contributions presented in the study are included in the article/supplementary material, further inquiries can be directed to the corresponding authors.
